# Comparison of Individual and Integrated Inline Raman, Near-Infrared, and Mid-Infrared Spectroscopic Models to Predict the Viscosity of Micellar Liquids

**DOI:** 10.1177/0003702820924043

**Published:** 2020-05-29

**Authors:** Kiran Haroon, Ali Arafeh, Stephanie Cunliffe, Philip Martin, Thomas Rodgers, Ćesar Mendoza, Michael Baker

**Affiliations:** 1 School of Chemical Engineering and Analytical Science, 5292 The University of Manchester, Manchester, UK; 2 Unilever R&D, Port Sunlight, UK

**Keywords:** Inline, viscosity, spectroscopy, near-infrared, NIR, mid-infrared, MIR, Raman, micellar liquids, partial least squares, PLS

## Abstract

In many industries, viscosity is an important quality parameter which significantly affects consumer satisfaction and process efficiency. In the personal care industry, this applies to products such as shampoo and shower gels whose complex structures are built up of micellar liquids. Measuring viscosity offline is well established using benchtop rheometers and viscometers. The difficulty lies in measuring this property directly in the process via on or inline technologies. Therefore, the aim of this work is to investigate whether proxy measurements using inline vibrational spectroscopy, e.g., near-infrared (NIR), mid-infrared (MIR), and Raman, can be used to predict the viscosity of micellar liquids. As optical techniques, they are nondestructive and easily implementable process analytical tools where each type of spectroscopy detects different molecular functionalities. Inline fiber optic coupled probes were employed; a transmission probe for NIR measurements, an attenuated total reflectance probe for MIR and a backscattering probe for Raman. Models were developed using forward interval partial least squares variable selection and log viscosity was used. For each technique, combinations of pre-processing techniques were trialed including detrending, Whittaker filters, standard normal variate, and multiple scatter correction. The results indicate that all three techniques could be applied individually to predict the viscosity of micellar liquids all showing comparable errors of prediction: NIR: 1.75 Pa s; MIR: 1.73 Pa s; and Raman: 1.57 Pa s. The Raman model showed the highest relative prediction deviation (RPD) value of 5.07, with the NIR and MIR models showing slightly lower values of 4.57 and 4.61, respectively. Data fusion was also explored to determine whether employing information from more than one data set improved the model quality. Trials involved weighting data sets based on their signal-to-noise ratio and weighting based on transmission curves (infrared data sets only). The signal-to-noise weighted NIR–MIR–Raman model showed the best performance compared with both combined and individual models with a root mean square error of cross-validation of 0.75 Pa s and an RPD of 10.62. This comparative study provides a good initial assessment of the three prospective process analytical technologies for the measurement of micellar liquid viscosity but also provides a good basis for general measurements of inline viscosity using commercially available process analytical technology. With these techniques typically being employed for compositional analysis, this work presents their capability in the measurement of viscosity—an important physical parameter, extending the applicability of these spectroscopic techniques.

## Introduction

Viscosity is the study of a material’s ability to flow and is regarded as the most important material characteristic.^1^ Measuring this property should therefore always be at the forefront of explorations into material deformation. The viscosity of many consumer products is determined not only by their fit for purpose criteria, but all sensory standards set out by consumers who judge the effectiveness of the products based on their consistency. For example, in the personal care industry, consumers expect a shampoo to be easily squeezed out of a bottle but one that is also thick enough to handle and distribute to their hair. Shampoo products that differ from these characteristics are assumed to be of inferior quality.

Process viscometry is a difficult task to achieve universally due to the criteria that apply to this type of instrumentation and the range and complexity of process fluids. Process instruments need to withstand exposure to hostile process conditions including plant vibrations, fouling, cleaning agents and dust, be simple in their operation, provide representative data (i.e., sample renewal should be fast and often) as well as measuring accurate and precise process specific data.^1,2^ Typically, compromises must be made when assessing potential instrumentation or techniques where the choice depends on the application.

Many industries could benefit from in situ technologies that are able to track the viscosity of products during the production process including food, paints/coatings, and pharmaceuticals who have numerous products where viscosity is a critical quality attribute as is the case in the personal care and cosmetics industry. Consequently, a few online and inline technologies have been marketed for this purpose, each with benefits and drawbacks related to their operation or implementation.^1,2^ Many of these technologies are based around principles of operation related to offline techniques such as the Brookfield TT100 based on traditional measurements of resistance to shearing such as benchtop rheometers/viscometers.

Measuring velocity profiles is a popular method of tracking viscosity during manufacture with techniques such as electrical resistance tomography (ERT) and ultrasonic velocity profiling (UVP) becoming increasingly prevalent.^3–7^ A large drawback of UVP is its necessity to add tracer particles to systems that do not contain reflective particles which is undesirable in strictly controlled environments. ERT has shown some promise in measuring the velocity profile of micellar liquids in a batch flow system where a relationship between conductivity and microstructure due to local shear rates was found.^3^ Even though the study shows the potential to monitor the velocity profile of micellar liquids, it is more complex to implement such a system in a production process.

Another study presented the measurement of in situ viscosity via decay of a fluorescence dye as a function of time. Major downfalls for this study are the health and safety issues concerned with the dye that was used, thioflavin T (ThT), which is described as an irritant and is corrosive, and toxic to humans and the environment. Another potential issue is the time needed to ensure the dye is well incorporated into the bulk, stated to be an hour for this study.^8^

Another fluorescence-based method uses synthetic fluorophores, known as molecular rotors, to measure microviscosity^9,10^ that could be used to measure viscosity inline. However, although there is a relationship between the bulk viscosity and microviscosity, it is unclear and subject to discussion amongst scientists.^11,12^ As with UVP, these fluorescence-based approaches would require the addition of fluorophores to the system which as stated is undesirable in a manufacturing environment.

Measuring heat transfer capacities as a means of indirect viscosity determination has also been explored by Schelden et al. for fermentation broths^13^ and Wunderlich et al. for non-Newtonian polymers.^14^ The work presented here was restricted due to the power output of the calibration heaters that poses issues when wanting to conduct pilot and small-scale operations, as the reactor volume needs to be large compared with the heat transfer area.

Malara et al.^15^ describe a recent and innovative in situ rheological measurement technique based on the vibration of a wire using a fiber Bragg grating sensor to allow for unparalleled strain sensitivity that cannot be rivalled by even the most sensitive benchtop rheometers. Although the technique shows potential, measurements are known to be affected by temperature and mechanical variation where the latter would be more of an issue in a manufacturing environment.

Due to their rapid, precise, and nondestructive nature, spectroscopic measurements play a key role in process analytical technology. They have the capacity to predict a range of chemical and some physical properties when combined with chemometric analysis. Some studies have been conducted on the use of near-infrared (NIR) and mid-infrared (MIR) to predict the viscosity of petroleum products where the NIR model shows superior performance.^16,17^ These studies show the potential for infrared spectroscopy to predict the viscosity of Newtonian liquids. Personal care liquids are seen to have shear thinning behavior,^18–21^ and are therefore non-Newtonian. Previous work in the literature has been found that uses infrared spectroscopy to predict the viscosity of non-Newtonian fluids, including pharmaceutical cream,^22^ gravy,^23^ chocolate,^24^ and latex.^25,26^ They all clearly show strong relationships between composition and viscosity where spectral variance is attributed to known compositional differences showing a distinct observable difference in NIR spectra. Data fused models have also been investigated to determine whether the predictive ability of the models can be enhanced using information from each of the techniques. Work combining NIR and MIR spectral information in predictive models has been explored for various applications including characterization of olives,^27^ Emmental cheese,^28^ and the oxidation of edible oils^29^ where in some cases the use of combined spectral data considerably improved the models, whereas in others NIR models performed best. It is interesting to note that across the range MIR models always showed the weakest performance.

Therefore, the objective of this work is to assess the use of vibrational spectroscopic techniques (NIR, MIR, and Raman) to measure the viscosity of micellar liquids in situ. This work builds on a previous study specifically looking at inline NIR spectroscopy for the measurement of viscosity of micellar liquids.^30^ In this work, the viscosity of the micellar liquid is dictated by the amount of salt present. The addition of salt affects the micelle structures formed by the surfactant molecules resulting in a change in viscosity. The electrolyte ions would act to shield the polar heads of surfactants reducing the repulsion between them and allowing them to pack closer together resulting in a more structured material with a higher viscosity. A few different microstructures are formed with continued addition of salt and follow the well-known salt curve, with worm-like micelles being the ideal structure. As the only differences in the samples used will be salt content and (water) at exceptionally low levels, the difficulty inheres in determining what the variation detected in the spectra/models is attributed to. No studies have been found relating to predicting viscosities using Raman spectroscopy. To the best of our knowledge no work has been found looking specifically at predicting the viscosity of micellar liquids using MIR or Raman spectroscopy. Data fused models will also be investigated to determine whether the predictive ability of the models can be enhanced using information from each of the techniques.

## Materials and Methods

### Samples

The samples were made up of 17% sodium lauryl ether sulfate (SLES), 5% cocoamidopropyl betaine (CAPB), and varying amounts of water and sodium chloride. The batch was produced with a formulation hole of 5.15% to adjust the viscosity of samples as necessary by varying electrolyte (NaCl) content (and water). Twenty samples ranging from 0.24% to 1.35% were included in the training set and four samples were kept for test set validation (0.50%, 0.74%, 0.79%, and 1.08%). Sample viscosities ranged from 1.36 to 36.78 Pa s, with validation samples in the range of 3.82–22.65 Pa s.

### Flow Setup

**Figure 1.**
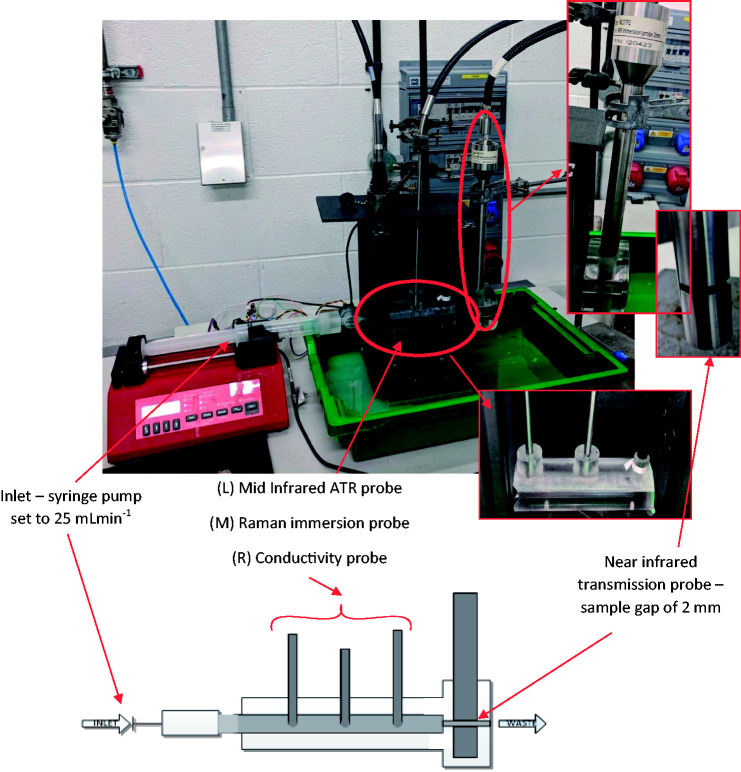
Experimental setup to simulate process conditions using a section of Perspex pipe (L = 20 cm, i.d. = 1.5 cm) designed to hold each of the inline process probes. Flow was simulated using a syringe pump set to 25 mL•min^–1^.

To simulate real process conditions on a small scale, a pipe specifically designed and built to hold all three spectroscopic probes was produced (Fig. 1). All probes are immersion probes, where the NIR probe operates under transmission mode, the MIR probe uses attenuated total reflection (ATR) and the Raman probe measures using backscattering. Therefore, the pipe was designed to ensure flow through the sample gap. A syringe pump was attached at the inlet and was set to produce a flow rate of 25 mL·min^−1^.

## Data Collection

### Near-Infrared Spectroscopy

The spectra were acquired with a Matrix F FTNIR (BRUKER, Germany) fiber coupled to a transmission process probe with a pathlength of 2 mm (Excalibur XP 20). The spectral range covered the whole of the NIR region (12 000–4000 cm^−1^). Spectra were acquired using a resolution of 8 cm^−1^ (2074 datapoints), averaged over 32 scans, with the samples at ambient temperature (≈25 ℃). Samples were measured twice successively using a background of air.

### Mid-Infrared Spectroscopy

The spectra were acquired with a Matrix MF FTIR (Bruker, Germany) fiber coupled to an ATR immersion probe (diamond crystal (IIIA), two reflections at 45 ℃ (IN350 T)). The setup for the inline MIR technique covered a spectral range of 3500–560 cm^−1^and spectra were acquired using a resolution of 4 cm^−1^ (1866 datapoints), 32 scans with an acquisition time of roughly 15 s and at ambient temperature (≈25 ℃). Each sample was tested twice successively using a background of deionized water. For comparative work between inline and offline MIR instruments, the Vertex 70 benchtop MIR spectrometer (Bruker, Germany) with a spectral range of 4000–400 cm^−1^ was used. This instrument also operates using ATR with a single reflection through a platinum diamond crystal. Offline spectra were acquired using a resolution of 4 cm^−1^ (2800 datapoints), 32 scans, and at ambient temperature (≈25 ℃).

### Raman Spectroscopy

The spectra were acquired with an RXNRaman1 system (Kaiser Optical Systems Inc.) fiber coupled to an immersion probe (IO 1/4″ [0.635 cm]S-NIR) using laser excitation at a wavelength of 785 nm. The spectrometer covered a range of 3425–150 cm^−1^. Each spectrum was acquired with an exposure time of 5 s and three accumulations (34 699 datapoints). Unlike the infrared spectroscopy, each sample was tested only once due to the longer time needed to obtain the measurement.

### Multivariate Analysis

Partial least squares regression was used to extract the useful data from the spectra and produce predictive viscosity models based on offline rheometer reference measurements. PLS Toolbox chemometrics software (PLS_Toolbox_8.0.1, Eigenvector Research Inc.) that runs in Matlab v.8.3 (The Mathworks Inc.) was used to develop these models.

For each technique, combinations of different pre-processing techniques were trialed to determine the best pre-treatment for each set of spectral data. Two baseline correction methods were trialed, detrending and Whitaker filter, and two scatter correction methods, standard normal variate (SNV) and multiple scatter correction (MSC), where the final step in all pre-processing operations was mean centering of the data. Spectra with no pre-processing applied (except mean centering) were also investigated, as viscosity changes in micellar solutions are consequential of microstructural changes. Variable selection was implemented using forward interval PLS (iPLS)^31,32^ where different window widths were trialed (5, 10, 30, 50, 100; +300 and 500 for Raman).

The root mean square error of cross-validation (RMSECV), prediction (RMSEP), and residual predictive deviation (RPD) were used to evaluate the predictive ability of the models and the coefficients of determination (R^2^_CV_, R^2^_Pred_) to determine the fit. Cross-validation was applied to the calibration samples using the venetian blinds method where samples were split into five subsets. It is important to note that log viscosity was used when developing the models to produce better fit. In order to better understand the performance of the models, predicted viscosities were converted back into Pa s and RMSEs recalculated.

The first step involved weighting the data sets which was done in one of two ways—either by using the signal-to-noise (S/N) for each technique or using the transmission curves for the infrared techniques. The noise in the spectra was calculated using the root mean square (RMS) method with a linear function defining the nominal signal. The S/N was then calculated using the calculated noise and the average signal over the defined spectral range. The regions used to calculate the S/N for the infrared techniques were the overall regions identified in the individual models (NIR: 727.9–11841.1 cm^−1^, MIR: 1008.7–1701.1 cm^−1^). The final S/N value was found by averaging that from each of the spectra in the data set (NIR: 4.5, MIR: 18.3). As the regions used in the individual Raman model are baseline regions showing no Raman shift, the value used to weight the Raman data was the average amplitude of the noise between 2580 and 2340 cm^−1^ where no peaks were present (Raman: 54.3). The weighted data were then subject to the pre-processing methods used in the individual models. Each data set was scaled using block variance scaling before being combined. The augmented data sets were then used to develop PLS models again using forward iPLS as the variable selection method.

Outlier analysis involved studying residual statistics (Q), Hoteling’s T^2^ statistics, and reviewing scores plots. Following the above analysis, one sample was found to be an outlier and was therefore removed from the calibration set.

### Reference Method

The viscosity of each sample was measured using a TA AR2000 Rheometer (TA Instruments, USA). Micellar liquids show shear thinning behavior using rotational rheometry; therefore, single-point measurements were taken at a shear rate of 0.4 s^−1^ (inside the Newtonian region) using a cone and plate geometry (diameter of 40 mm and angle of 2°). Samples were averaged over two measurements, temperature controlled (30 ℃) and covered to avoid drying during the experiment.

## Results and Discussion

### Spectral Analysis

**Figure 2.**
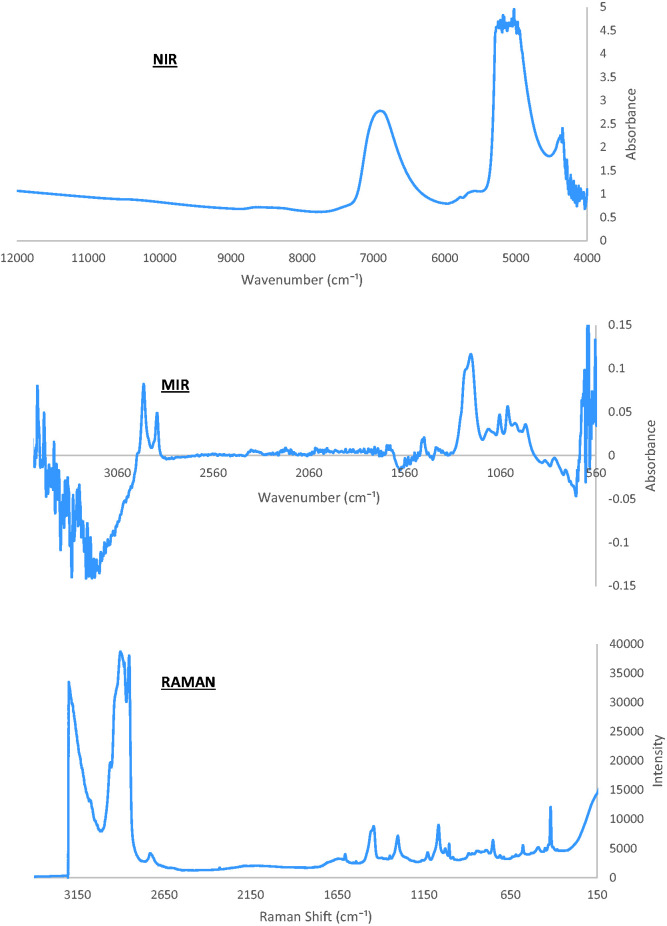
Spectral analysis of shampoo using NIR (above), MIR (middle), and Raman (below).

The main components present in the samples are SLES, CAPB, and water. A common structural feature for SLES and CAPB are their long alkyl chains which dominate in the NIR, MIR, and Raman spectra. Figure 2 shows a comparison of the spectra for each technique.

### Near-Infrared

Features present in the NIR spectra are the first and second overtones of C–H (5887–5457 cm^−1^ and 8797–7995 cm^–1^, respectively) and the second overtone of O–H (10537–9596 cm^–1^). The combination bands at the lower wavelengths (5366–4349 cm^–1^) and the first overtone of O–H (7427–6020 cm^–1^) both show intensities too high to be included in the calibration model. At these wavelengths, the transmission is low and noise is dominating.

### Mid-Infrared

The MIR spectra can be split into two: the main functional group region (4000–1500 cm^–1^), where peaks are isolated and easier to assign, and the fingerprint region (1500–400 cm^–1^), where the spectra becomes quite complex with many peaks present. The use of water as background eliminated the broad dominating water peaks allowing other components that may have been concealed as a result, to become better identifiable. The only peaks present in the main functional group region are related to CH_2_/CH_3_ stretching between 2957 and 2835 cm^–1^ and deformations between 1504 and 1412 cm^–1^ (wagging). The fingerprint region comprises of peaks related to the sulfonate and ether groups present in SLES. As a diamond crystal is used in the ATR probe, the spectra show regions of low S/N between about 2700 and 1500 cm^–1^ where transmission of light through the diamond is poor. Low S/N is also present further along between 3560 and 2700 cm^–1^ due to the transmission properties of the fibers.

### Raman

The acquired Raman spectra are dominated with strong peaks representative of CH_2_/CH_3_ stretching (3017–2645 cm^–1^) where other significantly smaller peaks can be observed relating to CH_2_ deformation, C–C skeletal stretching and SO_3_ stretches. Unlike MIR, features related to water are very weak in Raman spectroscopy and present no major peaks in these spectra.

## PLS Calibration Models

### Individual Models

#### Optimization of Wavenumber Region and Pre-Processing

The ideal pre-processing of the data will be different for each technique as they are subject to different spectral noise and variances unrelated to viscosity.

Raman spectra commonly encounter sample fluorescence resulting in intense background signals. These signals then become convoluted with the Raman signal and dominate the spectra making it difficult to analyze spectral regions that are a result of Raman scattering. These effects were minimized by use of a 785 nm excitation source and were not apparent in the spectra. To improve upon this, fluorescence effects can also be removed by means of baseline correction. In this work, detrending and use of a Whitaker filter were trialed. SNV has also been shown to be effective in removing noise in Raman spectra.^33,34^ Therefore, different combinations of baseline correction and SNV (followed by mean centering) were trialed.

In infrared spectroscopy, additive and multiplicative scatter effects commonly result in baseline variation between samples, which is seen in both NIR and MIR analysis, being more prominent in the NIR region. It is likely that due to the size of worm-like micelle structures present in the micellar liquids, that light is being elastically scattered in accordance with Mie theory where more forward scattering is experienced due to the size and shape of these micelles. Unlike some modeling algorithms, PLS is unable to implicitly account for multiplicative scattering affects; therefore, to minimize these effects scatter correction pre-treatment is required.^35^ Combinations of baseline correction (detrending) and scatter correction (SNV and MSC) techniques were trialed for infrared data sets. Eliminating the effect of scattering using the above techniques will likely remove information related to the microstructure of the samples. As viscosity changes in micellar solutions are due to microstructural changes, there may be some useful information in the scattering component of the spectra. Therefore, raw spectra with no preprocessing were also investigated during model development.

Trials were made using variable selection forward iPLS using different window widths (5, 10, 20, 30, 50; +100, 300, 500 for Raman) and different combinations of pre-treatments (as detailed above). The trials were judged based on RMSECV where the final variables and pre-processing techniques employed were those with the lowest RMSECV.

## Comparison of Models

**Figure 3.**
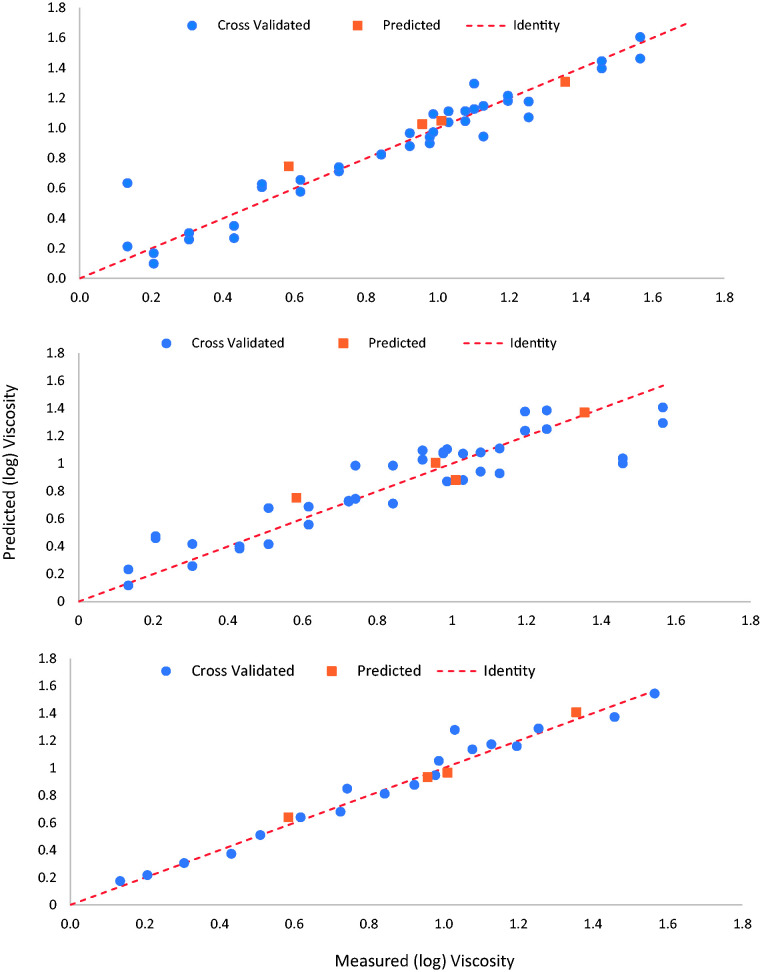
Correlation plots for individual predictive viscosity models using inline NIR (above), MIR (middle), and Raman (below). Plots show cross-validated predictions (circle) and test set validated predictions (square) where the dotted line represents the 1:1.

Details for all three individual optimized models are summarized in Table I including the optimized spectral range, rank (number of latent variables), RMSECV and RMSEP, and the RPD. Figure 3 presents the viscosity correlation plots for each model based on the training set data using cross-validation and test set data. Cross-validation was performed using the venetian blinds method, where the data set was split into five subsets leaving out 20% of the data for each cross-validation. MIR and NIR data contained two repeats of each sample (accounted for in the cross-validation), whereas Raman data consisted of one replicate due to time constraints. The test set data consisted of four samples that were not included in the development of the model.

**Table I.** Summary of statistics for individual NIR, MIR, and Raman predictive models.

	Near-infrared	Mid-infrared	Raman
Rank	2	7	9
RMSECV	2.51	5.90	2.45
R^2^_CV_	0.92	0.83	0.97
RMSEP	1.75	1.73	1.57
R^2^_PRED_	1.00	0.86	0.97
RPD_PRED_	4.57	4.61	5.07
Spectral regions (cm^–1^)	7717.9:7752.6 7833.6:7868.3 8026.5:8061.2 8180.7:8215.5 8257.9:8292.6 8412.2:8446.9 8682.2:8716.9 8759.3:8794.0 9762.1:9796.4 11343.5:11378.2 11806.3:11841.1	1008.7:1026.1 1105.1:1141.8 1375.2:1392.5 1587.3:1604.7 1683.7:1701.1	545.0:550.0 655.0:660.0 900.0:915.0 1370.0:1375.0 1780.0:1785.0 1980.0:1985.0 2020.0:2025.0
Total no. of variables	110	60	457
Pre-processing	None	SNV	Whitaker Filter/SNV
Scaling	Mean centered	Mean centered	Mean centered

RMSECV: root mean square error of cross-validation; RMSEP: root mean square error of prediction; RPD: residual predictive deviation; SNV: standard normal variate.

As mentioned previously, RMSEP is the best figure of merit for predictive ability of the model. For all three techniques, the RMSEP are comparable with the Raman showing the lowest (NIR: 1.75 Pa s, MIR: 1.73 Pa s, Raman: 1.57 Pa s). It is interesting to note that the cross-validation statistics differ significantly for all of the models (NIR: 2.51 Pa s, MIR: 5.90 Pa s, Raman: 2.45 Pa s). In terms of model complexity, the NIR model has the lowest rank of 2, whereas the Raman and MIR have a rank of 9 and 7, respectively.

The Raman and NIR models show good fit (R^2 ^= 0.97 and 1.00, respectively) with the MIR model having the worst fit (R^2 ^= 0.87). All RPD values are above 4 showing the capability of each model. However, the Raman model gives an RPD value over 5, suggesting this model would be more suited as a quality control measure than the infrared techniques. It is surprising that the MIR model performs poorly but this is likely due to the instruments bad S/N and poor transmission in the regions of greatest variance.

The NIR model was based on regions present in the second overtones of C–H and O–H, suggesting the model considers the evolving nature of the micellar network causing the differences in viscosity and the salt concentration of the samples. As the best model was developed using no preprocessing techniques, it is thought that the correlation relies strongly on microstructural information found in the scattering component. The variation detected in the C–H overtone is likely due to alignment changes of the lipophilic tails of the surfactant molecules. As more salt is added to the system, the repulsion between the polar heads of the surfactant molecules is reduced leading to tighter packing in the micelles resulting in alignment changes of the alkyl chains. The variation detected in the O–H overtone was at first thought to be representative of salt content as a few studies showed electrolyte content in aqueous solutions could be quantified using the O–H overtones in NIR spectra.^36–38^ However, a previous study showed that salt concentration was better represented using the second overtone of C–H for this data set.^30^ Therefore, the region of the second overtone of O–H may be more representative of water content as for this sample set the total water concentration decreased with increases in salt and so increases in viscosity.

All regions selected in the Raman spectra do not represent any specific peaks, they lie in areas between peaks or in areas showing no Raman shift suggesting the variance detected in these spectra could be related to background scattering effects as a result of microstructural changes that are affecting the viscosity as seen with the NIR model. However, modeling with raw Raman spectra showed a considerable decline in model performance.

For the MIR model, most of the regions selected are in the area of the spectra where S/N is very low due to poor transmission. Models based on regions included in this area would not be considered robust and would be prone to issues particularly when looking at calibration transfer. One other region selected is representative of the C–C skeletal stretch suggesting spectral changes are due to the changing alignment of alkyl chains of the surfactant molecules with increased addition of salt causing the change in viscosity which is being seen in the NIR model.

Previous work showed better models being produced using spectral regions on the edge of the MIR region of the spectrum, which are unattainable using the Matrix MF fiber coupled to the IN350 probe covering a range of 3500–560 cm^–1^. Using a benchtop ATR MIR instrument (Vertex 70, Bruker), the whole of the MIR region can be explored (4000–400 cm^–1^). A comparison between these two instruments was investigated looking at nine micellar liquid samples of varying viscosity. Optimized models were made using QUANT2 software (OPUS, Bruker). As only nine samples were used in this study, RMSECV was used to quantify the capability of these models.

The spectra from the benchtop instrument have significantly less noise; however, absorbances using the process probe are greater. This may be due to the path length of light for each crystal; the crystal in the inline setup is reflected twice, whereas the crystal on the benchtop instrument collects data from a single reflection. Between 2400 and 2300 cm^–1^, a doublet seems to be present in the spectra for both instruments; however, it is more prominent in the spectra obtained using the benchtop instrument. It was noted that this peak increased in intensity as subsequent measurements were made. The characteristics of this peak are typical of carbon dioxide in the atmosphere^39,40^ and can be avoided by performing backgrounds often or purging the system with an inert gas. For this work, the regions of interest are not affected by this peak and this peak can therefore be ignored.

Using QUANT2 software, an optimized predictive viscosity model was developed for the data obtained on the offline and inline instruments. QUANT2 can quickly produce an optimized model based on cross-validation (leave one out method). Similar to forward iPLS, the program used involved splitting the spectrum into 10 subregions and trialing combinations of subregions with the available pre-processing options until the final prediction error could not be further improved. The optimal model, as determined by the software, has the lowest RMSECV.

For the benchtop instrument, variations were found in three parts of the spectrum—the strongly absorbing CH_2_/CH_3_ stretches (2919–2558 cm^–1^), the absorbances related to S–O/SO_2_ and C–O–C stretches (1119–758 cm^–1^), and the last region being in the earlier part of the spectrum between 3639 and 3278 cm^–1^ showing a negative peak associated with water content, with frequencies that are not all available using the IN350. The region that is present in the spectrum using the inline setup (<3500 cm^–1^) is dominated by noise up to about 3200 cm^–1^ as the throughput of light is greatly reduced. The model developed using the inline data showed greatest variation on the edge of the spectrum (3276–2916 cm^–1^) roughly before the noise begins to dominate and includes part of the negative water peak and a part of the C–H stretches similar to the NIR model that used regions in the second overtone regions of O–H and C–H. The optimized models for the inline and offline gave very different RMSECV (5.37 Pa s and 1.58 Pa s, respectively), and more details on these models can be found in Table II.

**Table II.** Comparison of predictive viscosity models developed using benchtop and process MIR instruments.

Instrument	Matrix MF fiber coupled with IN350 ATR probe	Vertex 70
Type of measurement	Inline	Offline
R^2^ validation	0.33	0.94
RMSECV (Pa s)	5.37	1.58
Rank	2	3
Regions of interest (cm^–1^)	3276–2916	3639–3278 2919–2558 1119–758
Pre-processing	Constant offset elimination	Min–Max normalization

RMSECV: root mean square error of cross-validation.

## Integrated Models

Integrated data models were developed to determine whether combining data sets had any positive effects on model performance. To develop these models, the first step involved determining the best pre-processing techniques for each individual model (as highlighted in Table I). These were then applied to the whole spectrum for each technique. Data blocks (i.e., data associated with each technique) were then augmented in the variable direction. Block scale variance was used to account for the variation in the size of the signal for each technique; however, the variance in terms of block size (i.e., number of variables) was not accounted for. As with the individual models, forward iPLS was used to determine which parts of the integrated data set were the most useful for modeling viscosity.

To try to improve these models, it was thought to look at weighting the data sets in two ways: First by normalizing based on S/N ratio of each instrument. The second weighting technique is only applicable to the infrared techniques, using their transmission curves. The transmission curves detail the transmission efficacy at each frequency; therefore, the weighting procedure will put more emphasis on the frequencies showing good transmission properties. For both methods, the raw data sets were weighted prior to applying pre-processing techniques. It is important to note that unlike the individual NIR and MIR data sets which contained two replicates of each measurement, all augmented models contain one repetition of each sample to account for the Raman data which was only sampled once (due to time constrictions).

Table III presents the model statistics for these models including the number of variables included and the regions selected. Of the six combined models, three show improvements upon the predictive ability of the individual models. The best combined model consists of all the data sets weighted based on their S/N, with an error of prediction of 0.75 Pa s and an RPD of 10.62. However, it is the most complex model in this study made up of 10 latent variables. The next best model is the NIR–MIR model with an error of 1.24 Pa s; however, the region of the MIR spectra selected, like in the individual MIR model, lies in the part of the spectra showing poor transmission through the diamond and high levels of noise which is not representative of the spectra. The simple combined model including all three techniques also shows good performance with an RMSEP of 1.38 Pa s and a rank of 6.

**Table III.** Summary of model statistics for data-fused models.

Data sets	Weighting	Rank	No. of variables	RMSEP (Pa s)	R^2^_Pred_	RPD_PRED_	Spectral regions (cm^−1^)		
							Raman	NIR	MIR
NIR–MIR	–	4	50	1.24	0.998	6.47	–	4613.0:4628.4 5847.3:5862:7 5885.8:5901.2 9241.4:9256.9 9299.3:9314.7 9511.4:9526.9	1421.5:1429.2 2405.1:2422.4 2598.0:2605.7
NIR–Raman	–	2	20	2.73	0.989	2.93	2257.2:2257.6 2263.2:2264.1	9511.14:9526.8	–
MIR–Raman	–	1	20	7.02	0.32	1.14	47.1–48.0 62.1–63.0	–	None
NIR–MIR–Raman	–	6	50	1.38	0.981	5.81	226.1–228.0 1915–1916.0	8643.6:8678.3 11459.2:11493.9	None
NIR–MIR–Raman	Signal-to-noise	10	50	0.75	1	10.62	226.1:228.0 1915.1:1916.0	8643.6:8678.3 11459.2:11493.93	None
NIR–MIR–Raman	Transmission curves (MIR and NIR)	8	30	2.04	0.987	3.91	3300.1:3301.0	5712.3:5747.0 10032.1:10066.8	None

RMSEP: root mean square error of prediction; RPD: residual predictive deviation; NIR: near-infrared; MIR: mid-infrared.

The number of variables in the combined models is much lower than that of the individual models. The Raman data seems to dominate in most of the models and the MIR has the least contribution. This is representative of the individual models as the Raman model produced the lowest predictive error followed by the NIR model and the MIR model. However, as the Raman data had by far the largest number of variables, it is understandable that the Raman data had a substantial presence in each of the combined models. Is it likely that there may be some bias in terms of region selection due to this and future work should involve exploring ways to remove these effects. The regions selected for the combined models did not overlap with those used in the individual models. The regions of interest found in the NIR data varied, with some models focused on areas in the first overtone of C–H, others found information near the combination peaks and the most common region being at the higher frequencies in the second overtone regions of C–H and O–H, the only regions showing some consistency with the individual NIR model. The Raman contributions were concentrated in areas showing no Raman shift as seen with the individual model. The MIR data in the combined models is thought not to be useful. Having only contributed to the NIR–MIR model, the area selected in the MIR spectra lies in regions of low S/N, reducing the overall robustness of this model (as seen with some of the regions selected in the individual MIR model).

Overall, some of these integrated model statistics show considerable improvement in terms of prediction performance, though are countered in some cases by the increased complexity of the models and the use of spectral regions with low S/N. Another consideration, from an industrial standpoint, is that although the models may show enhanced performance, the implementation of two or more methods may not be practical or cost effective.

## Conclusion

This study aimed to develop predictive viscosity models for micellar liquids using three types of spectroscopy: NIR, MIR, and Raman. Successful models were built for each technique with all techniques showing comparable errors of prediction with Raman showing the lowest (NIR: 1.75 Pa s, MIR: 1.73 Pa s, Raman: 1.57 Pa s) and the MIR and NIR showing the best fit (R^2^_PRED_: Raman/NIR: 0.97, MIR: 0.83). For all models, it is thought that microstructural alignment changes are being detected. As more salt is added to the samples, the heads of the micellar networks can pack closer together, resulting in a change in positioning of the alkyl chains. In the NIR, this is indicated by variation detected in the second overtone of C–H. In the Raman analysis, the regions of interest lie between peaks in area showing no Raman shift. The MIR analysis shows variation around the C–C skeletal stretches, which again is likely to be related with the change in alignment of the alkyl chains.

The use of combined models was also explored and found to show some improvements in a few cases. The use of a simple combined model containing all three data sets produced a model with an improved RMSEP (1.38 Pa s) and a reasonable number of latent variables (six LVs). Weighting these data sets based on their S/N improved the model further reducing the RMSEP to 0.75 Pa s; however, this was offset with an increase in complexity of the model (10 LVs). The MIR data showed no real contribution to any of the combined models.

This work provides a good introduction into potential applications for spectroscopic process analytical technology in the personal care industry where viscosity is central to ensuring product quality. Based on implementation and model performance, NIR spectroscopy would be the best option. Although the Raman model showed slightly better performance in terms of error, implementing the technology would require more time and effort due to the hazards associated with the laser. Use of MIR showed comparable performance with a decreased fit and in terms of implementation would not be ideal in a factory setting as fiber optic cables would be limited to about 10 m.

As viscosity is an important parameter across numerous industries, this work provides an overview of potential inline spectroscopic techniques, highlighting the challenges that may arise when using each of the techniques and their effectiveness in predicting viscosity. As this work was based on viscosity changes of a single formulation due to electrolyte content, it will be useful moving forward to look at incorporating formulations containing different amounts of each surfactant. The amount of surfactants present and the ratio at which they are present will also affect the final viscosity and so by including different formulations in the model will ensure that the model is not just focused on detecting changes in salt content and will provide better insight into the extent of modeling the viscosity of micellar liquids. As the NIR probe operates in transmission mode, computational fluid dynamics will be used to determine whether entrance effects into the sample gap need to be considered. As mentioned previously, the Mie scattering component of the NIR data could provide information about the viscosity of the samples, and so trying to incorporate this knowledge into the model will be explored. Further work will also involve combining NIR data sets with other sensor measurements such as pressure drop to determine whether any improvements can be made to the predictive models.

## Declaration of Conflicting Interests

The author(s) declared no potential conflicts of interest with respect to the research, authorship, and/or publication of this article.

## Funding

The authors acknowledge funding and support from EPSRC and Unilever for an industrial case studentship (KH) and also for an EPSRC Prosperity Partnership with Unilever: Centre for Advanced Fluid Engineering and Digital Manufacturing (CAFE4DM) (EP/ R00482X/1).
